# Impact of Anatomical Resection on Non-transplantable Recurrence Among Patients with Hepatocellular Carcinoma: An International Multicenter Inverse Probability of Treatment Weighting Analysis

**DOI:** 10.1245/s10434-025-17349-y

**Published:** 2025-05-05

**Authors:** Jun Kawashima, Yutaka Endo, Mujtaba Khalil, Selamawit Woldesenbet, Miho Akabane, Andrea Ruzzenente, Francesca Ratti, Hugo Marques, Sara Oliveira, Jorge Balaia, François Cauchy, Vincent Lam, George Poultsides, Minoru Kitago, Irinel Popescu, Guillaume Martel, Ana Gleisner, Thomas J. Hugh, Luca Aldrighetti, Itaru Endo, Timothy M. Pawlik

**Affiliations:** 1 Department of Surgery, The Ohio State University Wexner Medical Center and James Comprehensive Cancer Center, Columbus, OH USA; 2 Department of Gastroenterological Surgery, Yokohama City University, Yokohama, Japan; 3 Department of Transplant Surgery, University of Rochester Medical Center, Rochester, NY USA; 4 Department of Surgery, University of Verona, Verona, Italy; 5 Department of Surgery, San Raffaele Hospital, Milan, Italy; 6 Department of Surgery, Curry Cabral Hospital, Lisbon, Portugal; 7 Department of HPB Surgery and Liver Transplantation, Beaujon Hospital, Clichy, France; 8 Department of Surgery, Westmead Hospital, Westmead, NSW Australia; 9 Department of Surgery, Stanford University, Stanford, CA USA; 10 Department of Surgery, Keio University, Tokyo, Japan; 11 Department of Surgery, Fundeni Clinical Institute, Bucharest, Romania; 12 Department of Surgery, University of Ottawa, Ottawa, ON Canada; 13 Department of Surgery, University of Colorado Denver, Denver, CO USA; 14 Department of Surgery, The University of Sydney, Sydney, NSW Australia

## Abstract

**Background:**

Among patients with hepatocellular carcinoma (HCC), the impact of anatomic resection (AR) versus non-anatomic resection (NAR) on non-transplantable recurrence (NTR) remains poorly defined. We sought to compare the risk of NTR among patients treated with AR versus NAR as the primary surgical strategy for HCC.

**Patients and Methods:**

Patients with HCC within Milan criteria who underwent curative-intent resection between 2000 and 2020 were identified from an international multi-institutional database. The inverse probability of treatment weighting (IPTW) method was utilized to compare short- and long-term outcomes among patients undergoing AR versus NAR.

**Results:**

Among 1038 patients, 747 (72.0%) patients underwent AR, while 291 (28.0%) patients underwent NAR. After IPTW adjustment, patients who underwent AR had better 5-year recurrence-free survival than individuals treated with NAR (63.9 vs. 52.0%; hazard ratio [HR] 0.78; 95% confidence interval [CI] 0.62–0.99); however, there was no difference in 5-year overall survival (80.2 vs. 75.6%; HR 0.76; 95% CI 0.55–1.05). Notably, individuals who underwent AR were less likely to have a NTR versus individuals treated with NAR (3-year NTR 9.8 vs. 14.4%; HR 0.62; 95% CI 0.40–0.96). In particular, AR was associated with a lower risk of NTR among patients with a medium tumor burden score (TBS) (HR 0.53; 95% CI 0.28–0.99), while the benefit among patients with a low TBS was less pronounced (HR 0.73; 95% CI 0.40–1.32).

**Conclusions:**

AR was associated with a lower risk of NTR and improved recurrence-free survival (RFS) among patients with HCC, especially individuals with higher TBS. An anatomically defined surgical approach should be strongly considered in patients with a higher HCC tumor burden.

**Supplementary Information:**

The online version contains supplementary material available at 10.1245/s10434-025-17349-y.

Hepatocellular carcinoma (HCC) is a leading cause of cancer-related mortality, being a major global health challenge with an increasing prevalence.^1,2^ Curative-intent treatment options include hepatic resection, liver transplantation (LT), and local ablation. However, only a small number of patients with early-stage HCC qualify for these treatment options.^3^ Hepatic resection is generally the preferred treatment option for patients with different stages of disease, provided liver function is well preserved.^4^ Whether anatomical resection (AR) versus non-anatomical resection (NAR) is the optimal surgical procedure for HCC has been a subject of debate.^5,6^ Given the lack of large-scale randomized controlled trials (RCTs), most studies comparing AR and NAR have been retrospective in nature.^7^ To minimize the inherent biases involved in comparing non-homogeneous cohorts, previous studies have utilized propensity scoring matching (PSM) to assess oncological outcomes relative to the two different surgical approaches.^5^ Several previous meta-analyses noted that AR for HCC was associated with better recurrence-free survival (RFS), while there have been conflicting results with respect to overall survival (OS).^5,8–10^

The incidence of recurrence, even after curative-intent resection of HCC, can be as high as 50–70%.^11,12^ Subsequently, repeat hepatectomy and local ablative therapies have been utilized as treatment options for recurrent HCC.^13^ In addition, salvage LT is an option to treat recurrent disease, having the benefit of eliminating any underlying intrahepatic tumor micro-metastasis theoretically reducing the risk of tumor recurrence.^14^ In a recent meta-analysis, salvage LT was noted to have a 1.36-fold greater survival benefit for patients with recurrent HCC compared with repeat hepatectomy.^14^ Unfortunately, roughly 40% of patients with HCC who undergo upfront liver resection develop a non-transplantable recurrence (NTR) ruling out the possibility of salvage LT.^13^ Although repeat liver resection is a treatment option for patients who are not candidates for salvage LT, repeat liver resection for NTR has been associated with a poor prognosis.^15^

While several studies have compared AR and NAR in terms of OS and RFS, the impact of AR versus NAR relative to the risk of NTR has not been examined.^5–10^ Therefore, the objective of the current study was to define the impact of initial AR versus NAR on the likelihood of patients to develop NTR among patients with HCC.

## Patients and Methods

### Study Population and Data Collection

Patients who underwent curative-intent hepatectomy for HCC between 2000 and 2020 were identified from an international multi-institutional database.^13^ Individuals who underwent curative-intent hepatectomy for primary HCC within Milan criteria were included in the analytic cohort.^16^ Patients who underwent palliative or R2 resection, as well as individuals with missing data on the surgical procedure or long-term outcomes, were excluded. Liver resection was categorized as AR when involving the systematic removal of Couinaud segment(s) encompassing the tumor, including the tumor-bearing portal vein and the associated hepatic territory.^5,7^ Conversely, a resection that did not adhere to the anatomical boundaries of liver segments was classified as NAR.^5,7^ The study was approved by the Institutional Review Board of each participating institution.

### Variables and Outcomes of Interest

Baseline covariates included patient age, sex, Charlson Comorbidity Index (CCI), year of surgery (i.e., 2000–2010 or 2011–2020), HCC etiology (i.e., hepatitis B or C, other), cirrhosis, Child–Pugh classification, albumin–bilirubin (ALBI) grade (i.e., grade 1 or grade 2/3), alpha-fetoprotein (AFP), and tumor burden score (TBS) on imaging. Surgical and pathological data included pathological margin status, the presence of microvascular invasion, histological tumor differentiation (i.e., well/moderately versus poorly/undifferentiated), post-hepatectomy complications, post-hepatectomy liver failure, and 90 day mortality. The ALBI score was calculated using serum albumin (g/L) and total bilirubin levels (mmol/L).^17^ TBS was computed using the formula: TBS^2^ = (maximum tumor diameter)^2^ + (number of tumors)^2^.^18^ Patients were categorized into two groups (low TBS: < 3; and medium TBS: ≥ 3 to < 9) as previously described.^18^ Major hepatectomy was defined as three or more segments according to the “New World” terminology.^19^ The severity of postoperative complications was defined according to the Clavien–Dindo classification system (grade I–V).^20^

The primary outcome of interest was NTR, which was defined as recurrence beyond the Milan criteria (i.e., single tumor > 5 cm in size, tumor number > 3, tumor number 2–3 but maximum tumor size > 3 cm, macroscopic vascular invasion, or extrahepatic disease).^13^ Secondary outcomes included RFS and OS. RFS was defined as the time between the date of liver resection and the date of HCC recurrence or death. OS was defined as the time between the date of liver resection and the date of death or the date of the last follow-up. After liver resection, patients were monitored every 3–4 months for the first 2 years, and every 6 months thereafter. During follow-up, patients were monitored by serum tumor markers (AFP) and imaging examinations (computed tomography and magnetic resonance imaging).^13^

### Statistical Analysis

Descriptive statistics were presented as median [interquartile range (IQR)] and frequencies (proportion, %) for continuous and categorical variables, respectively. Continuous variables were compared with the Mann–Whitney *U* or Kruskal–Wallis tests, as appropriate. Categorical variables were compared with the *χ*^2^ test or Fisher’s exact test, as appropriate. Multiple imputations with chain equations (MICE) procedures were employed to handle missing values.^21^

To balance the clinicopathological characteristics between the AR and NAR groups, inverse probability of treatment weighting (IPTW) was utilized on the basis of a propensity score (PS). The PS was estimated using logistic regression models predicting whether patients would undergo AR or NAR based on baseline variables (age, sex, CCI, year of surgery, etiology, cirrhosis, Child–Pugh classification, ALBI grade, AFP, and TBS). As for the IPTW, a pseudo population was created by weighting the inverse of the probability of a patient undergoing AR or NAR based on PS.^22^ The model preserved the size of the study population and no study participants were dropped (and statistical power lost), which was advantageous compared with the PSM method.^23^ Results of the comparison between co-variable subgroups were reported as standardized mean differences (SMDs). SMDs smaller than 0.1 indicated very small differences between means, whereas values between 0.10 and 0.30, between 0.31 and 0.50, and greater than 0.5 indicated small, moderate, and large differences, respectively.^24^ For subgroup analysis, patients were categorized according to TBS category (low and medium) and performed IPTW adjustments given that TBS has a high impact on NTR based on previously published data.^13,25^ PS was recalculated for the restricted population included in the subgroup analysis.

The OS, RFS, and NTR rates were calculated using the Kaplan–Meier method, and differences were compared using Cox proportional hazards analysis. Univariate and multivariate analyses of the baseline characteristics and preoperative clinical factors were performed using a Cox proportional hazards model after IPTW adjusting.

For sensitivity analysis, a multivariable Cox regression analysis for NTR was conducted using the unmatched cohort. All tests were two-sided, and a *p*-value < 0.05 was considered statistically significant. All statistical analyses were performed using R version 4.2.0 (R Project for Statistical Computing, Vienna, Austria).

## Results

### Baseline Cohort Characteristics

A total of 1038 patients were included in the analytic cohort. Median age at the time of surgery was 69 years (IQR: 61–74 years) and most patients were male (*n *= 794, 76.5%). The most common etiology of HCC was hepatitis B or C (*n *= 748, 72.1%). Roughly one-half of patients had ALBI grade 1 (*n *= 505, 48.7%), while only a small subset was classified as Child–Pugh B or C (*n *= 90, 8.7%). Moreover, median AFP was 11.0 ng/mL (IQR: 4.0–626.7). On preoperative imaging, most patients had a solitary lesion (*n *= 884, 85.1%) and median TBS was 3.0 (IQR: 2.7–3.1). On pathological assessment, 257 (24.8%) patients had microvascular vascular invasion, and a majority of the patients had a well/moderately differentiated tumor (*n *= 892, 85.9%). Moreover, a small subset of patients had positive resection margins (R1 resection) (*n *= 112, 10.8%). Overall, 315 (30.3%) patients experienced a postoperative complication, and 10 (1.0%) patients died within 90 days (Table 1).

**Table 1:** Comparison between patients undergoing anatomical resection versus non-anatomical resection before and after inverse probability of treatment weighting adjustment

		Unmatched cohort				IPTW-adjusted cohort			
	All patients	NAR	AR	SMD	*p*-Value	NAR	AR	SMD	*p*-Value
Variable	*n *= 1038	*n *= 291	*n *= 747			*n *= 1036.7	*n *= 1038.4		
Age, years, median (IQR)	69 (61, 74)	68 (60, 75)	69 (61, 74)	0.052	0.49	69 (60, 75)	69 (61, 74)	0.001	0.90
Sex, male, *n* (%)	794 (76.5)	217 (74.6)	577 (77.2)	0.063	0.40	798.1 (77.0)	794.5 (76.5)	0.011	0.87
CCI, median (IQR)	5 (4, 6)	5 (4, 6)	5 (4, 6)	0.031	0.28	5 (4, 6)	5 (4, 6)	0.015	0.79
Year of surgery, *n* (%)				0.119	0.09			0.004	0.95
2000–2010	371 (35.7)	116 (39.9)	255 (34.1)			369.1 (35.6)	371.9 (35.8)		
2011–2020	667 (64.3)	175 (60.1)	492 (65.9)			667.6 (64.4)	666.5 (64.2)		
Cirrhosis, *n* (%)	593 (57.1)	182 (62.5)	411 (55.0)	0.153	**0.03**	587.5 (56.7)	592.4 (57.0)	0.008	0.91
Etiology, *n* (%)				0.035	0.66			0.031	0.67
Hepatitis B or C	748 (72.1)	213 (73.2)	535 (71.6)			731.5 (70.6)	747.5 (72.0)		
Other	290 (27.9)	78 (26.8)	212 (28.4)			305.2 (29.4)	290.9 (28.0)		
Child–Pugh Score, *n* (%)				0.079	0.29			0.007	0.92
Class A	948 (91.3)	261 (89.7)	687 (92.0)			949.0 (91.5)	948.6 (91.4)		
Class B or C	90 (8.7)	30 (10.3)	60 (8.0)			87.7 (8.5)	89.8 (8.6)		
ALBI score, median (IQR)	−2.58 (−2.96, −2.11)	−2.56 (−2.94, −2.08)	−2.59 (−2.98, −2.13)	0.094	0.29	−2.58 (−2.94, −2.11)	−2.57 (−2.97, −2.11)	0.001	0.86
Grade 1, *n* (%)	505 (48.7)	136 (46.7)	369 (49.4)	0.053	0.48	506.4 (48.9)	501.1 (48.3)	0.012	0.86
Grade 2, 3, *n* (%)	533 (51.3)	155 (53.3)	378 (50.6)			530.3 (51.1)	537.3 (51.7)		
AFP, ng/mL, median (IQR)	11.0 (4.0, 626.7)	10.0 (4.0, 403.0)	11.0 (4.0, 698.5)	0.008	0.33	10.0 (3.0, 400.0)	12.0 (4.0, 698.1)	0.004	0.13
TBS, median (IQR)	3.0 (2.7, 3.1)	2.8 (2.5, 3.1)	3.1 (2.8, 3.2)	0.401	**< 0.001**	2.9 (2.7, 3.1)	3.0 (2.7, 3.1)	0.007	0.80
Low, *n* (%)	518 (49.9)	182 (62.5)	336 (45.0)	0.358	**< 0.001**	527.9 (50.8)	517.5 (49.8)	0.020	0.78
Median, *n* (%)	520 (50.1)	109 (37.5)	411 (55.0)			509.7 (49.2)	520.9 (50.2)		
Type of surgery				0.759	**< 0.001**			0.749	**< 0.001**
*Minor hepatectomy*	871 (83.9)	291 (100.0)	580 (77.6)			1036.7 (100.0)	811.1 (78.1)		
Non-anatomical resection	291 (28.0)	291 (100.0)	0 (0.0)			1036.7 (100.0)	0.0 (0.0)		
Single Segmentectomy	317 (30.5)	0 (0.0)	317 (42.4)			0 (0.0)	450.7 (43.4)		
Bi-segmentectomy/sectionectomy	263 (25.3)	0 (0.0)	263 (35.2)			0 (0.0)	360.5 (34.7)		
*Major hepatectomy*	167 (16.1)	0 (0.0)	167 (22.4)			0 (0.0)	227.3 (21.9)		
Right hepatectomy	86 (8.3)	0 (0.0)	86 (11.5)			0 (0.0)	115.1 (11.1)		
Left hepatectomy	60 (5.8)	0 (0.0)	60 (8.0)			0 (0.0)	82.4 (7.9)		
Extended right hepatectomy	7 (0.7)	0 (0.0)	7 (0.9)			0 (0.0)	9.8 (0.9)		
Extended left hepatectomy	9 (0.9)	0 (0.0)	9 (1.2)			0 (0.0)	13.1 (1.3)		
Central hepatectomy	5 (0.5)	0 (0.0)	5 (0.7)			0 (0.0)	6.9 (0.7)		
Complication, *n* (%)	315 (30.3)	82 (28.2)	233 (31.2)	0.066	0.38	306.4 (29.6)	324.2 (31.2)	0.036	0.61
PHLF, *n* (%)	59 (5.7)	17 (5.8)	42 (5.6)	0.009	1.00	53.8 (5.2)	58.9 (5.7)	0.021	0.75
90-day mortality, *n* (%)	10 (1.0)	3 (1.0)	7 (0.9)	0.010	1.00	10.4 (1.0)	9.5 (0.9)	0.010	0.88
MVI, *n* (%)	257 (24.8)	72 (24.7)	185 (24.8)	0.001	1.00	271.9 (26.2)	253.7 (24.4)	0.041	0.57
Histological grade, *n* (%)				0.055	0.49			0.058	0.42
Well to moderate	892 (85.9)	254 (87.3)	638 (85.4)			906.0 (87.4)	886.8 (85.4)		
Poor to undifferentiated	146 (14.1)	37 (12.7)	109 (14.6)			130.7 (12.6)	151.6 (14.6)		
Surgical margin, mm, median (IQR)	3.0 (1.0, 8.5)	4.0 (1.1, 9.0)	3.0 (1.0, 8.0)	0.007	0.44	3.2 (1.0, 8.0)	3.0 (1.0, 8.0)	0.022	0.60
R1 resection, *n* (%)	112 (10.8)	41 (14.1)	71 (9.5)	0.142	**0.04**	154.8 (14.9)	97.0 (9.3)	0.172	**0.01**

Bold font signifies a *p*-value < 0.05

*NAR* non-anatomical resection, *AR* anatomical resection, *IPTW* inverse probability of treatment weighting, *SMD* standardized mean differences, *CCI* Charlson Comorbidity Index, *ALBI* albumin–bilirubin, *AFP* alpha-fetoprotein, *TBS* tumor burden score, *PHLF* post-hepatectomy liver failure, *MVI* microvascular invasion

### Comparison of Clinicopathologic Characteristics Before and After IPTW

The total number of patients who underwent NAR and AR were 291 (28.0%) and 747 (72.0%), respectively (Table 1). Before IPTW adjustment, there were multiple imbalances between the two groups. Individuals who underwent AR were less likely to have cirrhosis (AR: *n *= 411, 55.0% versus NAR: *n *= 182, 62.5%; SMD = 0.153) and there was also a greater proportion of patients with medium TBS among patients who underwent AR (AR: *n *= 411, 55.0% versus NAR: *n *= 109, 37.5%; SMD = 0.358) than individuals who had a NAR. In addition, most patients underwent AR after 2011 (AR: *n* = 492, 65.9% versus NAR: *n *= 175, 60.1%; SMD = 0.119). After IPTW adjustment, the baseline characteristics of patients and tumors were closely balanced between the two groups (all SMDs < 0.100). In the IPTW-matched cohort, patients with AR were less likely to have a positive margin (AR: *n* = 97, 9.3% versus NAR: *n *= 154.8, 14.9%; SMD = 0.172). In addition, there was no difference in postoperative complications (AR: *n *= 324.2, 31.2% versus NAR: *n *= 306.4, 29.6%; SMD = 0.036) or 90 day mortality (AR: *n *= 9.5, 0.9% versus NAR: *n *= 10.4, 1.0%; SMD = 0.010).

### Survival After AR Versus NAR

**Fig. 1:**
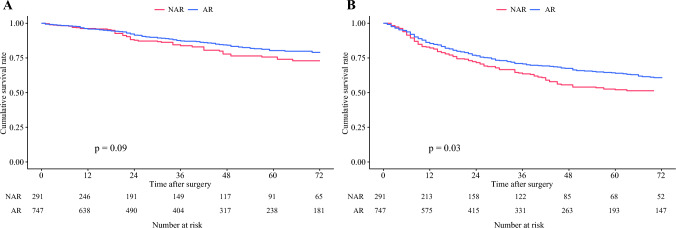
Kaplan–Meier curves demonstrating the differences in overall survival **A** and recurrence-free survival **B** stratified by patients who underwent anatomical resection (AR) and non-anatomical resection (NAR)

**Fig. 2:**
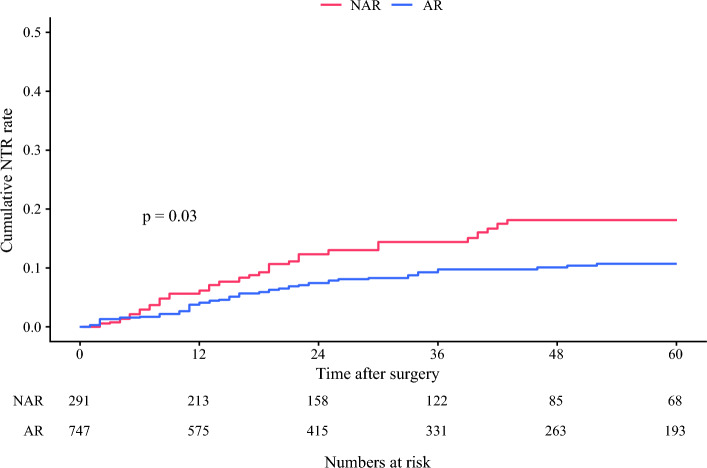
Cumulative non-transplantable recurrence (NTR) rate stratified by patients who underwent anatomical resection (AR) and non-anatomical resection (NAR)

The median patient follow-up was 38.0 months (IQR: 18.0–70.0). In the IPTW-matched cohort, patients who underwent AR had better 5-year RFS versus individuals who underwent NAR (63.9 vs. 52.0%; HR 0.78; 95% CI 0.62–0.99; *p *= 0.03); however, there was no difference in 5-year OS (80.2 vs. 75.6%; HR 0.76; 95% CI 0.55–1.05; *p *= 0.09) (Fig. 1). Notably, compared with patients who underwent NAR, individuals who underwent AR had a lower incidence of NTR (3-year NTR 9.8 vs. 14.4%; HR 0.62; 95% CI 0.40–0.96; *p *= 0.03) (Fig. 2). Similarly, in Cox regression analysis using the IPTW-matched cohort, after adjusting for patient baseline characteristics and tumor factors, patients who underwent AR had a markedly lower risk of NTR versus individuals who had NAR (HR 0.55; 95% CI 0.34–0.89; *p *= 0.01) (Table 2). This finding was also confirmed in a multivariable Cox regression model using the unmatched cohort (Supplementary Table 1).

**Table 2:** Cox regression analysis of demographic factors associated with non-transplantable recurrence after the inverse probability of treatment weighting adjustment

	Univariate analysis		Multivariate analysis	
Variables	HR [95% CI]	*p*-Value	HR [95% CI]	*p*-Value
Age	0.99 [0.97, 1.01]	0.41	0.99 [0.96, 1.02]	0.33
Sex, male	1.24 [0.67, 2.29]	0.49	1.22 [0.65, 2.29]	0.54
Charlson Comorbidity Index	1.07 [0.89, 1.29]	0.49	1.10 [0.87, 1.38]	0.43
Year of surgery				
2000–2010	Ref		Ref	
2011–2020	1.08 [0.64, 1.93]	0.77	1.03 [0.60, 1.77]	0.90
Etiology				
Hepatitis B or C	Ref		Ref	
Other	1.11 [0.64, 1.93]	0.71	1.03 [0.59, 1.82]	0.90
Cirrhosis	0.89 [0.55, 1.45]	0.65	0.94 [0.57, 1.56]	0.82
Child–Pugh class				
Class A	Ref		Ref	
Class B or C	0.59 [0.26, 1.36]	0.21	0.66 [0.28, 1.54]	0.33
ALBI grade				
Grade 1	Ref		Ref	
Grade 2, 3	0.73 [0.45, 1.18]	0.19	0.80 [0.48, 1.31]	0.37
AFP	1.00 [1.00, 1.00]	0.91	1.00 [1.00, 1.00]	0.86
Tumor burden score				
Low	Ref		Ref	
Medium	1.31 [0.82, 2.10]	0.25	1.28 [0.78, 2.12]	0.32
Anatomical versus non-anatomical resection				
Non-anatomical	Ref		Ref	
Anatomical	0.62 [0.40, 0.96]	**0.03**	0.55 [0.34, 0.89]	**0.01**
Minor versus major hepatectomy				
Minor hepatectomy	Ref		Ref	
Major hepatectomy	1.17 [0.69, 1.99]	0.56	1.59 [0.91, 2.77]	0.10

Bold font signifies a *p*-value < 0.05

*ALBI* Albumin–bilirubin, *AFP* Alpha-fetoprotein, *TBS* Tumor burden score

### Subgroup Analysis Stratified by TBS

**Fig. 3:**
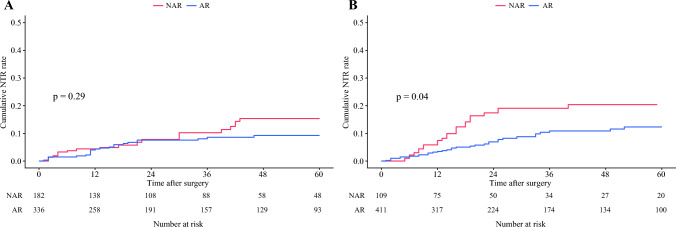
Subgroup analysis of cumulative non-transplantable recurrence (NTR) rate stratified according to tumor burden score (TBS) group: **A** cumulative NTR rates stratified by patients with a low TBS who underwent anatomical resection (AR) and non-anatomical resection (NAR); **B** cumulative NTR rate stratified by patients with a medium TBS who underwent AR and NAR

**Fig. 4:**
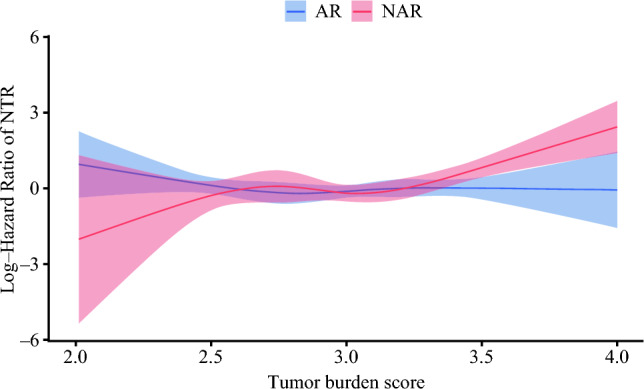
Hazard function curves for patients who underwent anatomical resection (AR) and non-anatomical resection (NAR) demonstrating a relationship with log hazard rate for non-transplantable recurrence (NTR) and tumor burden score

A total of 520 (50.1%) patients were categorized into the median TBS group, while 518 (49.9%) patients were categorized into the low TBS group. AR was performed in 411 (79.0%) patients with median TBS, and 336 (64.8%) patients with low TBS. In the IPTW-matched cohort, patients with a medium TBS score who underwent AR had a markedly lower risk of NTR versus individuals who underwent NAR (3-year NTR 10.9 vs. 19.1%; HR 0.53; 95% CI 0.28–0.99; *p *= 0.04). In contrast, among patients who had low TBS, the anatomic approach to hepatic resection was not associated with NTR risk (3-year NTR 8.6 vs. 10.2%; HR 0.73; 95% CI 0.40–1.32; *p *= 0.29) (Fig. 3). In the hazard function analysis of NTR, the hazard curve for patients who underwent AR was almost flat regardless of TBS value. Conversely, the hazard function curve for patients who underwent NAR increased with higher TBS values (Fig. 4).

## Discussion

Curative-intent treatment options for patients with early-stage HCC include liver resection or LT.^26^ LT may be a superior option for many patients with early-stage HCC, but its widespread use is restricted owing to a shortage of donor organs, the risk of tumor progression, and the possibility of patients dropping off the waiting list.^26^ As such, liver resection is the mainstay of curative-intent treatment of HCC.^5,6^ While liver resection is a well-established treatment option for HCC, the choice between AR and NAR remains a topic of debate.^27–30^ Theoretically, wider margins may improve disease control, whereas more limited resection preserves hepatic parenchyma and reduces the risk of liver insufficiency.^31^ As such, the ideal surgical approach should aim to optimize locoregional control while preserving as much non-tumorous hepatic parenchyma as possible.^8^ In addition, the surgical approach should reduce the risk of NTR, as treatment options for NTR are limited and mortality rates are high.^13–15^ To date, the risk of NTR following AR versus NAR remains poorly defined. The current study was important because it specifically defined the risk of NTR among patients who underwent AR versus NAR within the Milan criteria, leveraging data from a large international multi-institutional database. Notably, AR was associated with a lower risk of NTR after adjusting for patient and tumor characteristics using the IPWT method. In addition, among patients with a medium TBS, the risk of NTR was lower with AR versus NAR. Interestingly, while the benefit of AR relative to NTR was pronounced among patients with medium TBS, there was no benefit among patients with low TBS.

HCC recurrence after resection with curative intent can be as high as 50–70%, highlighting the importance of postoperative recurrence management.^13,26^ Of note, although all patients in the current study initially met Milan criteria at the time of liver resection, the 3-year risk of NTR—defined as recurrence beyond the Milan criteria—among individuals who underwent NAR was 14.4%. Treatment options for recurrent HCC include salvage LT, repeat hepatectomy, local ablation, and systemic therapy.^14^ Salvage LT was proposed by Majno et al. as an alternative option to primary LT for transplantable HCC.^32^ Notably, several meta-analyses have demonstrated that the prognosis of salvage LT is comparable to that of primary LT.^33,34^ Theoretically, salvage LT is a better treatment than other treatment strategies (i.e., repeat resection, ablation, etc.); specifically, LT eliminates any micro-metastases in the explanted liver, as well as removes the underlying cirrhotic background.^14^ In fact, a recent meta-analysis reported a 1.36-fold OS benefit with salvage LT versus repeat liver resection.^14^ In a separate study, 5‐year DFS and OS were both superior among patients who underwent salvage LT compared with individuals who underwent repeat liver resection (DFS, 71.6 vs. 32.8%, *p *<  0.001; OS, 72.8 vs. 48.3%, *p *= 0.007).^35^ Owing to donor shortages and favorable oncologic outcomes of salvage liver transplantation, some investigators suggested that salvage LT following primary liver resection was the optimal strategy for HCC.^34,36^ Unfortunately, approximately 40% of patients experience recurrence beyond Milan criteria and are not suitable candidates for salvage LT.^13^ In addition, liver resection of HCC beyond Milan criteria has been associated with a poor prognosis.^37,38^ In particular, Xing et al. reported that repeat hepatectomy for tumors larger than 5 cm increases the risk of mortality twofold.^15^ As such, reducing the risk of NTR is crucial to improve long-term outcomes of patients with HCC. While background and tumor characteristics are not mutable, surgeons can impact factors associated with the surgical procedure. In particular, decision-making regarding the surgical procedure (i.e., AR versus NAR) is modifiable. Importantly, data in the current study demonstrated that patients who underwent AR had a markedly lower risk of NTR compared with patients who underwent NAR.

Several previous meta-analyses had reported that AR for HCC was associated with better RFS.^5,8^ In one systematic review that included 43 studies with over 12,000 patients, Moris et al. reported that patients who underwent AR had improved 1-year, 3-year, and 5-year RFS compared with patients who underwent NAR.^8^ Similarly, a recent meta-analysis analyzing 22 PSM studies noted that AR was associated with a 1.2-fold lower likelihood of HCC recurrence versus NAR.^5^ Interestingly, both extrahepatic recurrence and multiple intrahepatic recurrences were lower in the AR group.^5^ Consistent with these data, the current study demonstrated that patients who underwent AR had a markedly lower risk of NTR. In terms of tumor pathology, two mechanisms are generally implicated as the causes of recurrence: intrahepatic and extrahepatic metastasis from the primary tumor; or intrahepatic metastasis from *de novo* multicentric tumor development.^11,39,40^ Recurrence from the primary tumor may be due to residual intrahepatic metastasis from the HCC spreading through the portal venous system, which cannot be detected before and during surgery.^41^ Several investigators noted that AR can mitigate and treat microscopic tumor spread along the portal vein, as well as remove peri-tumoral micrometastasis.^6,42^

While offering these theoretical benefits, whether AR is needed for all patients with HCC has been debated. Some investigators have suggested that AR might offer a survival benefit only in a subset of patients with specific tumor characteristics.^5,43^ For example, in a study from Japan, recurrence was lower among patients who underwent AR versus NAR, even those patients with tumors measuring 2–5 cm. However, there was no difference in recurrence for patients with tumors less than 2 cm.^43^ The size and number of tumors are known predictors of tumor recurrence and outcomes, with TBS recently being proposed as a comprehensive metric of tumor morphology that effectively stratified patients with HCC relative to prognosis.^18^ Existing literature has demonstrated the association of TBS with NTR.^13,25^ For example, Altar et al. demonstrated that the most important prognostic factor associated with NTR was TBS, highlighting the importance of tumor biology on recurrence patterns.^13^ Another study demonstrated that higher TBS was associated with a higher incidence of NTR, and patients with high TBS were more likely to recur earlier at an extrahepatic site.^25^ These studies suggested that LT should be considered instead of liver resection in patients with high NTR risk.^13,25,26^ In the current study, patients with a medium TBS had a higher incidence of NTR after NAR. In contrast, there was no difference in the risk of NTR following AR versus NAR among patients with a low TBS. The reasons for these disparate results may be due to a higher incidence of peri-tumoral micro-metastasis and portal tracking of tumors among patients with a higher TBS, which would make AR a better option. In turn, these data suggest that, while NAR may be acceptable for patients with a low TBS, AR should be the preferred surgical approach for patients with a medium TBS.

The results of the current study should be interpreted in light of several limitations. Given the retrospective nature of the study, there may have been residual confounding due to selection bias. While the use of an international, multi-institutional database was a strength, variability in patient selection, surgical techniques, and postoperative monitoring owing to varied protocols and criteria across different institutions may have existed. In particular, AR was performed on the basis of institution- and surgeon-specific protocols. The standardization of intraoperative methods, such as indocyanine green fluorescence imaging, intraoperative ultrasonography, or three-dimensional navigation, was not mandated. Consequently, variations in AR techniques may have influenced oncologic outcomes. In addition, institutional variability in postoperative monitoring may have influenced the timing of recurrence detection, potentially affecting the classification of NTR and RFS outcomes. Moreover, there may have been differences in selection criteria to perform AR versus NAR across different institutions and surgeons. Although IPTW analysis was employed to mitigate potential bias, only measured confounders can be accounted for using this statistical technique. Another limitation was the unavailability of data on the resected liver volume or weight. The database lacked information on intrahepatic recurrence location, preventing differentiation between local and remote intrahepatic recurrence. Consequently, we could not determine whether the observed effect was due to improved local disease control or underlying liver disease, although key liver function and background disease variables were adjusted using the IPTW approach. Another limitation involved our inability to assess high TBS, as patients with extensive tumor burden were often not candidates for either AR or NAR.

In conclusion, AR was associated with improved RFS and a lower risk of NTR among patients with HCC undergoing curative-intent resection. In particular, among patients with a higher TBS, AR provided better results than NAR with a low likelihood of NTR following the index hepatic resection. Thus, while NAR may be appropriate for patients with a low TBS, AR should be considered the preferred surgical approach for patients with a higher TBS when feasible.

## Supplementary Information

Below is the link to the electronic supplementary material.

Supplementary file1 (DOCX 45 KB)
