# Supplementary material for: Impact of Anatomical Resection on Non-transplantable Recurrence Among Patients with Hepatocellular Carcinoma: An International Multicenter Inverse Probability of Treatment Weighting Analysis
Source: Ann Surg Oncol. 2025 May 5;32(9):6243–53. doi: 10.1245/s10434-025-17349-y (PMC12317867; doi:10.1245/s10434-025-17349-y)
Supplement: Supplementary file 1 — Supplementary file1 (DOCX 45 KB) [file 10434_2025_17349_MOESM1_ESM.docx]

**Supplementary Table 1.** Cox regression analysis of demographic factors associated with non-transplantable recurrence using the unmatched cohort.

|  | Multivariate analysis | |
| --- | --- | --- |
| Variables | HR 95%CI | *P* value |
| Age | 0.97 [0.95, 0.99] | **0.002** |
| Sex, male | 1.46 [0.86, 2.48] | 0.16 |
| Charlson comorbidity index | 1.21 [1.06, 1.38] | **0.004** |
| Year of surgery |  |  |
| 2000-2010 | Ref |  |
| 2011-2020 | 1.19 [0.77, 1.85] | 0.43 |
| Etiology |  |  |
| Hepatitis B or C | Ref |  |
| Other | 1.09 [0.69, 1.71] | 0.71 |
| Cirrhosis | 0.89 [0.59, 1.36] | 0.59 |
| Child-Pugh class |  |  |
| Class A | Ref |  |
| Class B or C | 0.77 [0.35, 1.70] | 0.51 |
| ALBI grade |  |  |
| Grade 1 | Ref |  |
| Grade 2, 3 | 0.82 [0.53, 1.25] | 0.35 |
| AFP | 1.00 [1.00, 1.00] | 0.23 |
| Tumor burden score |  |  |
| Low | Ref |  |
| Medium | 1.17 [0.77, 1.76] | 0.46 |
| Anatomical vs. non-anatomical resection |  |  |
| Non-anatomical | Ref |  |
| Anatomical | 0.61 [0.39, 0.97] | **0.03** |
| Minor vs. Major hepatectomy |  |  |
| Minor hepatectomy | Ref |  |
| Major hepatectomy | 1.57 [0.91, 2.71] | 0.10 |

^ALBI, Albumin-bilirubin; AFP, Alpha-fetoprotein; TBS, Tumor burden score; Bold font signify p-value < 0.05^
